# Supplementary material for: Role and mechanism of NCAPD3 in promoting malignant behaviors in gastric cancer
Source: Front Pharmacol. 2024 Apr 22;15:1341039. doi: 10.3389/fphar.2024.1341039 (PMC11070777; doi:10.3389/fphar.2024.1341039)
Supplement: Supplementary file 11 [file DataSheet2.ZIP › GSEA/Oncogenic signature/my_analysis.Gsea.1599461962066/MEK_UP.V1_DN.html]

Details for gene set MEK\_UP.V1\_DN[GSEA]

|  || Dataset | filtered\_dataset.sample\_info.cls#WT\_versus\_NCAPD3\_MUT |
| Phenotype | sample\_info.cls#WT\_versus\_NCAPD3\_MUT |
| Upregulated in class | WT |
| GeneSet | MEK\_UP.V1\_DN |
| Enrichment Score (ES) | 0.11423049 |
| Normalized Enrichment Score (NES) | 0.53927714 |
| Nominal p-value | 0.95652175 |
| FDR q-value | 0.961079 |
| FWER p-Value | 1.0 |
Table: GSEA Results Summary

  

Fig 1: Enrichment plot: MEK\_UP.V1\_DN      
 Profile of the Running ES Score & Positions of GeneSet Members on the Rank Ordered List

  

| SYMBOL | TITLE | RANK IN GENE LIST | RANK METRIC SCORE | RUNNING ES | CORE ENRICHMENT || 1 | 54894 | RNF43 | 29 | 1.000 | 0.0493 | Yes |
| 2 | 323 | APBB2 | 35 | 0.977 | 0.1142 | Yes |
| 3 | 22834 | ZNF652 | 164 | 0.711 | 0.0716 | No |
| 4 | 6672 | SP100 | 185 | 0.689 | 0.1055 | No |
| 5 | 9315 | NREP | 364 | 0.557 | 0.0160 | No |
| 6 | 54665 | RSBN1 | 429 | 0.516 | 0.0060 | No |
| 7 | 9830 | TRIM14 | 486 | 0.480 | -0.0008 | No |
| 8 | 23255 | MTCL1 | 505 | 0.471 | 0.0193 | No |
| 9 | 5378 | PMS1 | 506 | 0.471 | 0.0523 | No |
| 10 | 90806 | ANGEL2 | 583 | 0.434 | 0.0279 | No |
| 11 | 23586 | DDX58 | 584 | 0.433 | 0.0583 | No |
| 12 | 7290 | HIRA | 612 | 0.417 | 0.0681 | No |
| 13 | 56339 | METTL3 | 613 | 0.417 | 0.0973 | No |
| 14 | 178 | AGL | 711 | 0.368 | 0.0531 | No |
| 15 | 3434 | IFIT1 | 743 | 0.354 | 0.0556 | No |
| 16 | 4071 | TM4SF1 | 851 | -0.251 | -0.0041 | No |
| 17 | 5723 | PSPH | 859 | -0.267 | 0.0096 | No |
| 18 | 51141 | INSIG2 | 903 | -0.328 | 0.0016 | No |
| 19 | 3665 | IRF7 | 956 | -0.375 | -0.0097 | No |
| 20 | 9926 | LPGAT1 | 1036 | -0.434 | -0.0363 | No |
| 21 | 253959 | RALGAPA1 | 1066 | -0.456 | -0.0253 | No |
| 22 | 26053 | AUTS2 | 1113 | -0.485 | -0.0244 | No |
| 23 | 6947 | TCN1 | 1135 | -0.503 | -0.0043 | No |
| 24 | 27250 | PDCD4 | 1170 | -0.537 | 0.0088 | No |
| 25 | 9665 | KIAA0430 | 1192 | -0.566 | 0.0334 | No |
| 26 | 8345 | HIST1H2BH | 1207 | -0.585 | 0.0644 | No |
| 27 | 1475 | CSTA | 1400 | -1.162 | 0.0072 | No |
Table: GSEA details [plain text format]

  

Fig 2: MEK\_UP.V1\_DN      
 Blue-Pink O' Gram in the Space of the Analyzed GeneSet

  

Fig 3: MEK\_UP.V1\_DN: Random ES distribution      
 Gene set null distribution of ES for **MEK\_UP.V1\_DN**
